# Barriers and facilitators to recruitment of physicians and practices for primary care health services research at one centre

**DOI:** 10.1186/1471-2288-10-109

**Published:** 2010-12-13

**Authors:** Sharon Johnston, Clare Liddy, William Hogg, Melissa Donskov, Grant Russell, Elizabeth Gyorfi-Dyke

**Affiliations:** 1 Department of Family Medicine, University of Ottawa, 43 Bruyère Street, Ottawa, Ontario, Canada; 2 CT Lamont Primary Health Care Research Centre, Élisabeth Bruyère Research Institute, 43 Bruyère Street Ottawa, Ontario, Canada; 3 Bruyère Continuing Care, 43 Bruyère Street, Ottawa, Ontario, Canada; 4 Department of Family Medicine, University of Ottawa, 43 Bruyère Street, Ottawa, Ontario, Canada; 5 School of Primary Health Care, Monash University, Notting Hill, 1/270 Ferntree Gully Rd, Melbourne, Victoria, Australia; 6 Institute of Population Health, University of Ottawa, 1 Stewart Street, Ottawa, Ontario, Canada

## Abstract

**Background:**

While some research has been conducted examining recruitment methods to engage physicians and practices in primary care research, further research is needed on recruitment methodology as it remains a recurrent challenge and plays a crucial role in primary care research. This paper reviews recruitment strategies, common challenges, and innovative practices from five recent primary care health services research studies in Ontario, Canada.

**Methods:**

We used mixed qualitative and quantitative methods to gather data from investigators and/or project staff from five research teams. Team members were interviewed and asked to fill out a brief survey on recruitment methods, results, and challenges encountered during a recent or ongoing project involving primary care practices or physicians. Data analysis included qualitative analysis of interview notes and descriptive statistics generated for each study.

**Results:**

Recruitment rates varied markedly across the projects despite similar initial strategies. Common challenges and creative solutions were reported by many of the research teams, including building a sampling frame, developing front-office rapport, adapting recruitment strategies, promoting buy-in and interest in the research question, and training a staff recruiter.

**Conclusions:**

Investigators must continue to find effective ways of reaching and involving diverse and representative samples of primary care providers and practices by building personal connections with, and buy-in from, potential participants. Flexible recruitment strategies and an understanding of the needs and interests of potential participants may also facilitate recruitment.

## Background

In our rapidly changing primary health care system, practice-based research is essential to guide quality improvement and reform [1]. Recruitment of providers and practices to participate in primary care research, however, is a major challenge in the drive to develop primary care based knowledge. While there has been some research examining the challenges and opportunities in recruitment of physicians and patients for primary care research [2-12], further research on recruitment methodology is needed, given that this remains a challenging and crucial aspect of primary care research.

Practice-based research is a vital part of advancing primary care knowledge. Recruitment for research participation is a critical component of generating high quality evidence. However, participation rates in primary care practice-based studies vary greatly [2] and can threaten how representative the findings may be. The literature on research recruitment in primary care suggests several strategies research teams might employ to improve recruitment rates such as, the use of physician recruiters [2], involving a local champion - usually a physician [9], and minimizing the burden of participation on the practice [2]. While health care providers recognize the importance of research [2,4,5,11,13,14], barriers to participation for community-based providers are substantial [2]. Inhibiting factors such as lack of time, lack of capacity, interruption of patient flow, and lack of familiarity or understanding of research objectives [2,13,15] will remain, unless systematic efforts are taken to address these factors by research teams, providers, professional associations and training programs, health care funders and other stakeholders interested in generating a primary care evidence base. Therefore, methods to increase research participation need more attention from the research community. In addition, the effect of ongoing primary care restructuring and reform on the participation of providers in research needs to be addressed.

This paper shares the results of a quality assessment project undertaken to understand which factors facilitated or hindered recruitment of physicians and primary care practices for several large scale current, or recently completed, health services research projects conducted in one primary health care research centre in Ontario, Canada. Comparing approaches and outcomes for recruitment offers insight into some common challenges in recruitment that still need to be overcome.

## Methods

In order to improve recruitment methods, a quality assessment project was initiated to collect data on recruitment methods, results, and challenges. This data was collected from a convenience sample of five different primary care health services research studies conducted between 2004 and 2010 by investigators at the Élisabeth Bruyère Research Institute, University of Ottawa, Canada. All five projects received approval from the institutional Bruyère Continuing Care Research Ethics Board. The projects were all initiated as part of a major national effort to improve primary care and, thus, took place during periods of significant primary care reform. A Principal Investigator for each project was approached and six individuals, three Principal Investigators and three Project Coordinators, who were not part of the quality assessment project team, had a semi-structured interview with EG-D about recruitment methods used, results, challenges encountered and innovative practices used. A Project Coordinator for each study completed a short survey on recruitment methods, results, and challenges. See Additional file 1 for an outline of the survey topics, which guided the semi-structured interviews. See Table 1 for a brief description of each project.

**Table 1:** Description of primary care projects

Project	Description
**COMP-PC *(Comparison of Models of Primary Health Care in Ontario Project) ***(2004-2006)	**Summary**: This study took place in Ontario, Canada between 2004 and 2006. This mixed methods study was designed to compare the quality of primary care services delivered in four predominant service delivery models in Ontario, Canada: Fee for service (FFS); Family Health Networks (FHNs); Health Services Organizations (HSOs); and Community Health Centres (CHCs). (Details about these models of services delivery are available elsewhere) [19]. **Type of Research**: Cross-sectional mixed methods study. **Data collection**: tools included provider, patient, and practice surveys, chart audits, and in-depth interviews with select providers, patients, and key informants.

**IDOCC *(Improved Delivery of Cardiovascular Care Program) ***(2007-current)	**Summary**: This study is currently underway in the Champlain district of Ontario, Canada. This program is intended for primary health care providers to improve the delivery of evidence-based prevention and management strategies for diabetes, heart disease, and stroke within their practice. **Type of Research**: Stepped wedge randomized controlled trial. **Data collection**: includes chart abstraction, and the program includes monthly customized visits from facilitators.

**ICFPC *(I Care for Primary Care) ***(2004-2005)	**Summary**: This completed project, started in October 2003, involved 30 practices in Southern Ontario and Ottawa, Canada. This was an outreach facilitation program intended to improve the delivery of preventative care, with a chronic disease care management component. **Type of Research**: Before-and-after trial. **Data collection**: the program included chart reviews, patient surveys, and meetings with physicians every 3-4 weeks to discuss prevention issues.

**CHAP *(Cardiovascular Health Awareness Program) ***(2001-current)	**Summary**: Started in 2001, this was a multi-phase project in 20 communities in Ontario, Canada focused on cardiovascular health awareness of seniors. The program involved engaging physicians to recruit their patients, through various methods, to cardiovascular risk assessment sessions in local pharmacies. **Type of Research**: Clustered randomized community intervention trial. **Data collection**: Physicians received data from these sessions that they could then use for patient follow-up. Physicians were also asked to complete a survey following the completion of the project.

**FWS *(Financial and Work Satisfaction) ***(1999-2006)	**Summary**: This study took place in Ontario, Canada, examining potential changes (e.g. on incomes, workloads, and work satisfaction) for physicians who entered into primary care reform. **Type of Research**: mixed method study (cross sectional and cohort study). **Data collection**: included collecting physician income data from tax records, as well as survey and demographic data.

A summary of each of the five included studies is provided including: relevant time frame, location, and study design.

Data analysis used a mixed-methods approach. Descriptive statistics for each study were generated from the survey results. Qualitative analysis was based on a thematic analysis of interviewees' responses. One team member (EG-D) reviewed her interview notes, and prepared a summary table comparing answers across each question. Key points arising from the interviews, such as common experiences arising in two or more of the projects, issues deemed important to a project, disconfirming experiences, or innovative approaches employed to overcome common challenges were noted and shared with two other team members, (SJ and WH) for review and discussion. Survey answers were reviewed separately by another team member (SJ) to identify common, important, or disconfirming experiences. Both data sets were then compared by one team member (SJ) to identify explanatory theories and data which supported these theories. Emerging themes were then compared to trends previously identified in a literature review for confirmation or disconfirmation.

## Results

An overview of the projects' details, including sampling frame, recruitment methods, participation rates and challenges is presented in Table 2. While there were many similarities in methods, participation rates ranged from 20% to 49%. Data from these practice-based studies have been grouped into the themes of barriers and facilitators to successful recruitment of physicians and practices.

**Table 2:** Recruitment information for projects

Project	Participant Information	RECRUITMENT METHODS				Recruitment Rate	Representative Advisory Group	Incentives	Time Required to Recruit	Flexible Strategies
		**Dillman/Modified Dillman**	**Use of Local Opinion Leader**	**Face to Face Recruiters**	**Responsible for Recruitment**					
							
**COMP-PC **(2004 - 2006)	Practices: 137 recruited All CHC, HSO, & FHN in Ontario +197 randomly selected FFS-FHG Providers: 363 (surveyed) 46 (interviewed) Patients: 5361 (surveyed) 24 (interviewed)	Modified Dillman (invitation material mailed and combination of methods for follow-up)	Yes - limited to end of study - regional leaders made phone calls	Yes - response rate much higher-done only at end of study	Practice recruitment by investigators with support from central organizations. Practices invited eligible providers. Patients recruited through front desk.	Avg Practices (45%) of 365 eligible practices. 82% of 6522 eligible patients Provider participation not tracked if >50%	Yes - very useful. Had stakeholder advisory group at least 2 members of each group studied	*Financial: *$2,000 for quantitative component -$500 for qualitative *Non Financial: *Token gifts for period of data collection	9 months - targets not reached entirely	Invitation letters, follow up letters, emails & telephone calls. Finally, face to face recruitment

**IDOCC **(2007 - current)	Practices: Approached = 372 Recruited = 93 Physicians: Approached = 1077 Recruited = 200	Modified Dillman (invitation material mailed and combination of methods for follow-up)	Step 1 - yes Step 2 - no	Yes - project staff (outreach facilitators) visited providers who had not responded - challenging strains on physician time	Lead physicians were sent letters and asked if interested in IDOCC. Physician investigator made follow-up phone call if necessary.	Avg Practices (25%) of 372 eligible practices. 19% of 1077 eligible physicians.	Yes - stakeholder advisory group	*Non Financial*: Assistance with practice improvement	Step 1 - almost 10 months Step 2 - 4 months	No

**ICFPC **(2004 - 2005)	Practices: Eligible = 99 Recruited = 30 Physicians: Eligible = 164 Recruited = 58	Modified Dillman (invitation material mailed and combination of methods for follow-up)	Yes	No	Contact made through lead physician at each site. If interested all other physicians were contacted.	Avg Practices (30%) of 99 eligible practices. 100% of eligible physicians (58 of 58 physicians from recruited practices).	No	No	9 months	No

**CHAP **(2001 - current)	Communities: Identified = 41 Eligible = 39 Participated = 39 20 Communities in CHAP average 17.1 physicians. 19 Communities in Control average 19.1 physicians.	No (Information session, physician meeting, letters & follow-up)	Yes - physician opinion leader model used	Yes - local opinion leaders used at start - called community meeting - local coordinators traveled to physician office	Opinion leader physicians used to recruit colleagues	Physicians (49%) of approximately 700.	Yes, in some areas, not everywhere	*Non Financial: *Feedback on individual participants sent directly to physicians	Unsure	Yes

**FWS **(1999 - 2006)	Family Physicians recruited	Modified Dillman	No	No	No	Physicians (20.2%)	No	No	Repeat mailings and reminders (5 in total)	No

This table provides relevant recruitment information for each of the studies used in the sample. Responses are based on surveys of Investigators and Project Coordinators from each of the projects.

### Barriers to recruitment

#### Sampling frame

Each of the studies had similar sampling frames and approached potential physician participants through their practices to seek participation of the practice, the practice and individual practice members, or individual providers. Three studies adopted the practice and affiliated providers as the sampling units, while the other two recruited family physicians. Two study investigators reported that their studies encountered significant difficulties in creating a sampling frame due to the lack of reliable and up-to-date information on primary care providers and practices. The longitudinal study IDOCC reported investing annually in updating the list of practices eligible to participate in its study. The COMP-PC study found that ongoing health care reform initiatives resulted in a significant flux in models of primary care delivery, as practices and providers changed models leading to the ineligibility of 10% of the initial sample approached. Further, the lack of available information required project staff to invest additional time during the screening for eligibility and recruitment phases to clarify models of service delivery. This information was difficult to collect from the practices and often could not be obtained from front-office staff.

The time required for recruitment efforts varied across the studies. Despite sample sizes ranging from 30 practices to 137 practices, and a wide range in recruitment rates, most studies required over nine months to recruit participants. Furthermore, most of the studies invested more time and effort into recruitment than originally anticipated.

#### Front-office rapport

A major recruitment challenge for many of the projects was engaging a practice receptionist to relay the study information to the physician who would ultimately decide whether or not the selected practice would participate. To remedy this challenge, three of the five studies sent project staff to the practices to recruit in person. The IDOCC study used nurse practice change facilitators to recruit. The CHAP study, which had the highest recruitment rate at 49%, was the only project to use face-to-face recruitment from the onset of the study. CHAP also found innovative ways to engage front-office staff early, such as holding a luncheon presentation for support staff, which outlined the proposed research project. The other four studies all used an initial mailed invitation to the physicians at their site of practice.

### Facilitators to recruitment

#### Flexible strategies

Most projects employed a Dillman, or modified Dillman, approach to recruitment [16]. This involved an initial mailout from the research team to potential participants, usually physicians, followed by a second mailout or fax, and then a telephone call. Tactics that were used to increase recruitment rates through this approach included personalizing the correspondence, having the physician investigators sign the letter, and having physician investigators make direct contact. However, two of the most successful recruitment efforts, for the CHAP and COMP-PC projects, adopted flexible recruitment strategies allowing them to adapt to the local culture and engage the assistance of available community resources such as community agencies, local opinion leaders, and professional organizations. For example, the COMP-PC project found fee-for-service practices to be more challenging to recruit than other models. However, maintaining a flexible approach to recruitment allowed the COMP-PC research team to make adjustments to the recruitment strategy in order to overcome the initial poor participation of fee-for-service practices.

One alternative strategy in recruitment, which was used by the CHAP project to raise awareness of cardiovascular health issues among seniors in smaller communities, engaged 20 community organizations in the recruitment of local family physicians and pharmacists [17]. Coordinators within these organizations were asked to identify and work with 'opinion leaders', that is, those physicians and pharmacists who were well-regarded at the local level. These opinion leaders championed participation in the CHAP project to their colleagues and clients. Strategies to promote CHAP included distributing personalized letters, speaking at hospital rounds, displaying posters, circulating information sheets, and offering word-of-mouth referrals to patients and pharmacy clients [17]. The lead community organization and recruitment efforts varied depending on the resources, needs, and experience of the community.

#### Incentives

Three of the five projects offered participation incentives. However, only one study, COMP-PC, which had the second best recruitment rate of 45%, provided direct financial incentives of up to $2500 per practice. There was also a wide variation in indirect incentives for participating practices including, practice change management assistance, feedback on patients' health outcomes and practice processes, continuing medical education credits and other resources for the practice, such as resource binders. Many of the investigators and coordinators felt that the indirect incentives were equally or more important to the participants than financial incentives. Several investigators and coordinators also identified the relevance of the research question as an important feature for recruitment, as professionals and practices were motivated to participate when they believed the study would provide them with useful and practical information.

#### Characteristics of the recruiter

Four projects employed project staff members to do the majority of the contacting and coordinating related to recruitment. The CHAP project used local physician opinion leaders as well as staff from the lead local community organizations to assist with recruitment. However, the IDOCC project initially employed a similar strategy to that of CHAP, and had one of the lowest recruitment rates. Several investigators and coordinators noted that the staff recruiter becomes the representative for the project and, thus, must employ excellent interpersonal skills when making initial contact with potential participants. In effect, the success of the recruitment efforts for many of the studies was dependent upon the recruiter's ability to liaise with individuals at potential practice sites and establish an initial personal rapport.

Though several studies reported that it was not feasible for physicians to do all the recruiting, the experience across many of the five studies was that the physician investigators did still play an important role in shaping and adapting recruitment efforts throughout the study. The COMP-PC project reported that strong and regular support and direction from senior project staff and investigators was essential to enable the recruitment coordinator to adapt the recruitment strategy to demands and emerging challenges. Physician investigators also became involved at later stages in the recruitment process, for example, to make follow-up calls to selected practices after other strategies had failed to produce satisfactory participation rates.

#### Participant buy-in and perceived relevance

Several projects enhanced participant buy-in by highlighting the relevance of the project to potential participants. All of the five studies except FWS used local opinion leaders or representative advisory groups to endorse the research study and several allowed study investigators to directly approach potential participants they knew. The advisory groups could provide legitimacy and profile to the projects, thereby enhancing trust in the research agenda among their colleagues. For example, in the COMP-PC project, endorsements from recognized leaders and advisory committee members, as well as an endorsement and publicity on a provincial association's website, helped highlight the importance of the research to providers and patients. The FWS study, one of the more sensitive projects which involved examining physicians' tax records, employed focus groups with physicians to assist in the development of the recruitment letter. This was viewed as helpful as it ensured that the most appropriate wording was used in the recruitment letters in order to encourage maximum participation.

## Discussion

Our findings, from five large practice-based primary care health service research studies, highlight the investment required for recruitment of physicians and practices. Despite the wide range in participation rates, the studies shared common barriers to recruitment and addressed them in different, and sometimes innovative, ways. The noted barriers and facilitators to recruitment highlight two key challenges for recruitment: 1) the importance of making an effective personal connection with potential participants and 2) the importance of building participant buy-in.

### Recruitment for research participation: creating a personal connection

The success of the recruitment for these studies was dependent upon the ability of the research teams to create a personal connection with potential participants. While the research teams conducting these studies were aware of the evidence on recruitment strategies, the most successful strategies outlined in the literature, were often not feasible. Direct recruitment of physicians by physicians, and the use of personal connections, have been shown to increase recruitment rates [2]. However, recruitment rates achieved with direct physician-to-physician contact range from 33% to 90% [2,4,10]. In addition, as the results of this study have indicated, some research studies require buy-in from practice staff as well as multidisciplinary team members, making professional connections less effective.

In this sample, the projects that used physician recruiters for their studies believed this was a key factor in successful recruiting. However, the differences between participation rates do not seem to be explained by the use of physician recruiters. As physician recruiters are not always feasible, research teams can build flexible recruitment strategies, as the two most successful studies in this assessment did, and find creative ways to direct limited physician resources to recruiting priorities. As more primary care practices involve multi-disciplinary teams, non-physician providers might offer assistance in the recruitment process as the practice facilitators did for the IDOCC project. Additionally, the use of local organizations to recruit is also a way to create personal connections meaningful to members of that particular community.

The research teams' experiences in these studies also highlight the importance of engaging front-office members of primary care teams in the recruitment process. Following the traditional mail-outs and phone calls, face-to-face recruiters, ranging from project staff, to local community organization volunteers, were used by several projects. This approach could be effective in creating personal relationships with front-office staff. While face-to-face recruitment has been found to be more cost-effective than mail-out invitations [3], this approach might be less cost-effective in studies encompassing large geographic regions. A flexible recruitment strategy might allow in-person recruitment, should other methods fail.

None of these projects involved a Practice Based Research Network (PBRN). While practices in a PBRN have already signaled an interest in research participation and may have a pre-existing relationship with research centres, they still must be recruited into any individual study. Many of the challenges in recruitment experienced by the studies assessed in this project have also been described for work with PBRNs [18].

### Successful recruitment requires participant buy-in

Recent literature on recruitment has highlighted the need to sell the benefits of a study to the practice or physicians, and not just the merits of the research [10,19]. The findings from the five studies reviewed suggest that recognition and reward for providers' time in research projects may be increasingly important. Non-financial incentives were felt to be important in most of the studies' recruitment.

All five projects involved health services research, which may be viewed differently by physicians than clinical trial studies. Clinical trials may have built-in incentives as they offer patients new, better, or otherwise unavailable care [12]. However, in an era of greater focus on performance measurement and accountability, health services research may also be able to offer participants valuable incentives through the sharing of practice-level or physician-level performance findings. More attention to understanding the needs of current primary care practices might allow research teams to build into their protocols results-sharing mechanisms, which might assist in attracting participants.

In this study, investigators reported that the perceived relevance of their research was a factor in recruitment success. The two most successful studies for recruitment had representative advisory groups. Indeed, building participant buy-in requires efforts from research teams to create relevant research proposals. Partnering with organizations with which potential participants are affiliated, such as malpractice insurance organizations, HMO's, and professional organizations, may help promote the value of the research to primary care [7,12]. However, the culture and environment of family medicine is often less supportive of research participation than other specialties [3,20]. Greater efforts to involve professional and leading community organizations in supporting the research agenda might assist in promoting participation.

Finally, valuable research team time was used to create accurate and up-to-date lists of potential participants during this period of accelerated primary care reform. Many other primary care health services research studies have relied on lists of providers created by professional organizations. Partnering with other key stakeholders such as government health ministries, regional health authorities, or insurance companies, interested in promoting evidence-based practice might help address the lack of reliable information available to researchers. During times of rapid organizational change in primary care delivery these stakeholders might invest in the creation of updated databases of practices and providers to be made available to researchers with relevant projects [21]. This would also facilitate time-sensitive research on issues important to the community such as in situations of emerging pandemics.

Traditionally, recruitment protocols are designed in advance [6] based on varying degrees of evidence [5] and remain static throughout to ensure minimal bias in the sample. Different models of practice were found to present distinct challenges in recruitment, a finding reported in other studies[2,10]. The rapidly changing nature of the primary care delivery environment in many countries may herald the need for more flexible recruitment strategies that can be adapted to emerging realities, marked variety in governing structures and practice patterns within a sample, and unique community resources or professional organizations. Using scarce resources, such as physician recruiters, strategically for regions or types of practice which present initial lower participation rates, might allow for more successful and efficient recruitment. Ultimately research teams need to be innovative in seeking to create personal connections with all potential participants in addition to building participant buy-in. Successful recruitment may increasingly require that research teams engage with and learn about their target participants. Whether dealing with multiple layers of accountability for large groups, or poorly resourced solo practices, research teams might better anticipate where to involve more direct physician recruiters or staff capable of connecting with practice members.

### Limitations

All five studies were conducted in roughly the same time period of widespread primary care reform in a single health care system and conducted solely or primarily from a single academic centre. As this was an internally initiated review of recruitment practices in a research centre, several of the authors were involved with the studies reported as Principal or Co-Principal Investigators (WH-COMP-PC, IDOCC, ICFPC, FWS; CL: IDOCC), as a Co-Investigator (GR: COMP-PC), or as Project Manager (MD: COMP-PC). However the studies recruited well outside the region or sphere of influence of the investigators. The studies also recruited across a wide variety of practice models and rural as well as urban regions. The trends occurring in their settings are similar to widespread changes throughout the developed world. Nonetheless, local factors and individual leaders may have a strong impact on recruitment, limiting the generalizability of the experience of these studies.

## Conclusion

Practice-based research is essential to generate certain types of knowledge that are relevant to the actual context in which most patients receive their primary care. As health reform initiatives pilot new ways of organizing and delivering care, it remains essential to engage in practice-based research and evaluation. A main finding from across the five studies is that recruitment of providers and practices requires a significant investment of time and may need flexible strategies, in order to allow research teams to change their approach or methods as they learn from experience throughout the recruitment process. This study indicates that the challenge will be for investigators and project staff to remain adaptable while promoting the representativeness of the sample ultimately recruited.

## Competing interests

The authors declare that they have no competing interests.

## Authors' contributions

SJ and WH conceived the study. SJ, CL, MD and EG-D collected data. SJ, CL, WH, GR and EG-D analyzed the data. SJ and EG-D wrote the manuscript. WH, CL, MD and GR edited the manuscript. All authors read and approved the final manuscript.

## Pre-publication history

The pre-publication history for this paper can be accessed here:

http://www.biomedcentral.com/1471-2288/10/109/prepub

## Supplementary Material

Additional file 1 **Project Coordinator survey for study information**. Additional file 1 outlines the survey topics sent to each project team and which guided semi-structured interviews with Investigators and/or designated Project Coordinators for each study. Click here for file
