# Supplementary material for: Barriers and facilitators to recruitment of physicians and practices for primary care health services research at one centre
Source: BMC Med Res Methodol. 2010 Dec 13;10:109. doi: 10.1186/1471-2288-10-109 (PMC3017524; doi:10.1186/1471-2288-10-109)
Supplement: Additional file 1 — Project Coordinator survey for study information. Additional file 1 outlines the survey topics sent to each project team and which guided semi-structured interviews with Investigators and/or designated Project Coordinators for each study. [file 1471-2288-10-109-S1.PDF]

Additional File 1: Project manager survey for project information

	**COMP-PC**	**IDOCC**	**ICFPC**	**CHAP**	**FWS**
Recruitment rate					
Sampling frame					
Participants					
Dillman/Modified Dillman					
Recruitment correspondence addressed to specific individuals					
Focus group of target participants consulted for recruitment methodology					
Use of local opinion leader to recruit					
Flexible recruitment strategies responding to community					
Physician recruiter					
Representative advisory groups					
Encountered challenges engaging front office to share info with physicians. Please comment.					
Face to face recruiters					
Financial incentive to practices- amount					
Non-financial incentives					
Data collection at single or multiple time points					
How many recruited were subsequently not eligible based on exclusion criteria. %					
Time required to recruit					
Total budget spent on recruitment or % of budget					
